# Pre-Clinical Evaluation of a ^213^Bi-Labeled 2556 Antibody to HIV-1 gp41 Glycoprotein in HIV-1 Mouse Models as a Reagent for HIV Eradication

**DOI:** 10.1371/journal.pone.0031866

**Published:** 2012-03-09

**Authors:** Ekaterina Dadachova, Scott G. Kitchen, Gregory Bristol, Gayle Cocita Baldwin, Ekaterina Revskaya, Cyril Empig, George B. Thornton, Miroslaw K. Gorny, Susan Zolla-Pazner, Arturo Casadevall

**Affiliations:** 1 Albert Einstein College of Medicine, Bronx, New York, United States of America; 2 AIDS Institute, Division of Hematology/Oncology, The David Geffen School of Medicine at University of California Los Angeles, Los Angeles, California, United States of America; 3 Pain Therapeutics, Inc., Austin, Texas, United States of America; 4 Department of Pathology, New York University School of Medicine, New York, New York, United States of America; 5 Veterans Affairs New York Harbor Healthcare System, New York, New York, United States of America; University of California San Francisco, United States of America

## Abstract

**Background:**

Any strategy for curing HIV infection must include a method to eliminate viral-infected cells. Based on our earlier proof-of-principle results targeting HIV-1 infected cells with radiolabeled antibody (mAb) to gp41 viral antigen, we embarked on identifying a suitable candidate mAb for preclinical development.

**Methodology/Principal Findings:**

Among the several human mAbs to gp41 tested, mAb 2556 was found to have high affinity, reactivity with multimeric forms of gp41 present on both the surface of virus particles and cells expressing HIV-1 Env, and recognition of a highly conserved epitope of gp41 shared by all HIV-1 subtypes. Also, mAb 2556 was the best in competition with HIV-1+ serum antibodies, which is an extremely important consideration for efficacy in the treatment of HIV patients. When radiolabeled with alpha-emitting radionuclide 213-Bismuth (^213^Bi) - ^213^Bi-2556 efficiently and specifically killed ACH-2 human lymphocytes chronically infected with HIV-1, and HIV-1 infected human peripheral blood mononuclear cells (hPBMCs). The number of binding sites for ^213^Bi-2556 on the surface of the infected cells was >10^6^. The in vivo experiments were performed in two HIV-1 mouse models – splenic and intraperitoneal. In both models, the decrease in HIV-1 infected hPBMCs from the spleens and peritoneum, respectively, was dose-dependent with the most pronounced killing of hPBMCs observed in the 100 µCi ^213^Bi-2556 group (P = 0.01). Measurement of the blood platelet counts and gross pathology of the treated mice demonstrated the lack of toxicity for ^213^Bi-2556.

**Conclusions/Significance:**

We describe the preclinical development of a novel radiolabeled mAb reagent that could potentially be part of an HIV eradication strategy that is ready for translation into the clinic as the next step in its development. As viral antigens are very different from “self” human antigens - this approach promises high selectivity, increased efficacy and low toxicity, especially in comparison to immunotoxins.

## Introduction

Any strategy for curing HIV infection must include a method to eliminate viral-infected cells. This basic fact has been recognized for almost two decades. Despite the success of HAART (highly active antiretroviral therapy) in effectively reducing the viral burden of HIV to essentially undetectable levels, the occurrence of viral blips and the rebound of virus levels upon cessation of treatment suggests a long-lived reservoir of latently infected cells [1], [2]. HIV-1 latency is believed to represent a major obstacle to achieving a curative AIDS therapy. This becomes even more paramount as the HIV/AIDS population ages due to the success of HAART. Drug resistance, compliance issues, the financial burden of care and the inability of HAART to fully restore health have brought about a renewed focus on finding a cure for HIV/AIDS [3], [4].

One approach to addressing the HIV infected cell population that persists in the presence of HAART is to directly target and kill HIV-1 infected cells by using HIV-specific antibodies that specifically recognize cell surface expressed HIV-1 proteins (e. g. gp120/gp41) to deliver a toxic moiety, such as a cytotoxin (immunotoxin) or a radionuclide. Although immunotoxins were introduced as early as 1988 as potential HIV-1 drugs [5] and have been the subject of continuous improvements for the treatment of AIDS and cancer [6], [7], they still have inherent drawbacks that are impossible to overcome, including immunogenicity which precludes their repeated use; the need for internalizing antibodies; the necessity to target every single diseased cell to eliminate the disease; the need for complex chemistry; and instability with potential toxin-mediated collateral damage [7], [8]. In addition, any HIV eradication strategy will have to face the challenge of low or absent expression of viral antigens such as gp41/gp140 on the surface of latently infected cells [1]–[4] which will have to be overcome by application of viral reactivation agents. We anticipate that any effort to eradicate HIV-1 would require multiple cycles of depletion of viral infected cells followed by viral reactivation followed by renewed depletion of viral-infected cells. Hence, we need a strategy for depletion of viral-infected cells that is specific, relatively non-toxic and that can be used multiple times.

Radioimmunotherapy (RIT) uses tumor antigen-specific monoclonal antibodies (mAbs) for targeted delivery of cytocidal ionizing radiation to the tumor cells [9]–[11]. The distinct advantages of RIT are its relative independence on the immune status of the patient and not being a subject to drug resistance mechanisms, with both of these features being very useful in the management of HIV-infected patients. Indeed, the multiple transporters on the cells that are capable of pumping out small molecular chemotherapeutic drugs do not affect the antibody binding to their respective targets on the cell surface and subsequent killing of the cells by the ionizing radiation. The antibodies used in RIT are non-neutralizing and thus cannot put a selective pressure on the virus. Finally, the epitopes on the viral proteins chosen for RIT are conserved throughout the HIV strains and clades which suggest the importance of their maintenance in the viral Env and, as a result, will more than likely be present even on the mutated virions and consequently, on the HIV-infected cells. Historically, RIT has been used as an anticancer strategy. In this traditional approach, cancer RIT targets self antigens that are preferentially expressed on the tumor cells. We have demonstrated that RIT also has broad potential for the treatment of fungal and bacterial infections through targeting microbial antigens with radiolabeled mAbs in experimental models of fungal and bacterial infections [12], [13]. Consequently, we expanded RIT strategy to the HIV field and showed that HIV-1 infected cells were eliminated in vitro and in vivo by targeting gp120 and gp41 viral proteins expressed on the surface of infected cells with radiolabeled specific mAbs to these proteins [14]. In contrast to immunotoxins, in which a mAb is conjugated to immunogenic toxin, RIT does not elicit immune responses to radiolabeled human mAbs (as both a mAb and an isotope are not immunogenic) and is highly versatile given the numerous radionuclide options available. RIT is already an established therapeutic modality in oncology [11] with radiolabeled mAbs being approved for treatment of primary, recurrent and refractory non-Hodgkin lymphoma (NHL). Hence a logistical capacity exists in advanced care hospitals for delivering RIT and we anticipate that this infrastructure can be adapted for the eradication of HIV-1.

Following proof-of-principle experiments on using RIT for eradication of HIV-1 infected cells [14], we have now identified a human mAb known as 2556 as our lead candidate for preclinical development of RIT for HIV-1 eradication. MAb 2556 is a human mAb that recognizes a conserved region of HIV-1 gp41 and is less vulnerable to interference from endogenous antibody responses to HIV-1 than other anti-gp41 mAbs. Here we describe the initial selection and characterization of mAb 2556, the evaluation of clinical grade mAb 2556 in vitro, and efficacy and safety studies of clinical grade mAb 2556 radiolabeled with α-emitting radionuclide 213-Bismuth (^213^Bi) in SCID mice infected intrasplenically or intraperitoneally with HIV-1 infected human peripheral blood mononuclear cells (hPBMCs).

## Materials and Methods

### Selection of mAb 2556

Human mAb 2556 was initially produced from the hPBMCs of an HIV-1 infected individual living in Cameroon who provided signed consent prior to donating blood. Ethical clearances for production of human mAbs have been approved by the National Ethical Committee of Cameroon and the IRB of NYU School of Medicine. The infecting virus was not isolated from this blood sample, however, it is assumed to be non-subtype B, as subtype B viruses are not present in Cameroon. A standard cellular method based on fusion of Epstein-Barr virus-transformed lymphocytes with heteromyeloma cells was used in our laboratories to generate mAb 2556 [15]. The 2556 heterohybridoma cell line was established and produced an IgG1 lambda anti-gp41 mAb.

#### Epitope mapping and relative affinity

MAb 2556 (NYU, New York, NY) was tested by ELISA against overlapping gp41 peptides representing the consensus sequences of group M, subtype B and subtype C (NIH AIDS Research and Reference Reagent Program). MAb relative affinity was determined by measuring the concentration of a mAb needed to achieve 50% of maximal binding to recombinant gp41_MN_ by ELISA.

#### Competition study with serum antibodies

A binding assay was used to determine the ability of antibodies to gp41 in patients' sera to compete with mAbs to gp41 for binding to virus infected cells. These experiments were undertaken because serum from every HIV-1 patient contains polyclonal antibodies against gp41, which may potentially compete with human mAbs to gp41 in binding to HIV-infected cells. In this study, 293T cells (ATCC, Manassas, VA) were transfected separately to express HIV-1 Env from DJ 263.8 (CRF02_AG), JR-CSF (clade B), and MW965 (clade C). The transfected cells were exposed to biotinylated mAbs 2556 (NYU, New York, NY), 50–69 [16] and 246 (also known as 246D) [17], in the presence of serially diluted serum from HIV-1 infected individuals.

#### Binding to virus-transfected cells and to intact virions

To determine the cross-reactivity of mAb 2556 and other anti-gp41 mAbs, the binding assay was employed with 293T cells expressing Env of one of 15 T-cell lab adapted (TCLA) viruses and primary isolates representing subtypes A, B, C, D and CRF02_AG. The study compared the binding of mAb 2556 and two other mAbs to gp41, mAbs 246 and 50–69, mAb 1570 against CD4-binding domain of gp120 [18], and the negative control human mAb 1418 against parvovirus B19 [19]. Using the virus capture assay, as described in [20], mAb 2556 and two mAbs to gp41, 50–69 and 246 were tested to determine the extent of cross-reactivity with 42 intact viruses representing TCLA and primary isolates from several HIV-1 subtypes, A, B, C, D, F, G, H and CRF02_AG.

### Manufacturing of clinical grade 2556 mAb

2556 hetero-hybridoma cells were transferred from NYU to Aragen Biosciences, Morgan Hill, CA where the mRNAs for the heavy and light chains (HC and LC, respectively) of the 2556 mAb were isolated, reverse transcribed and cloned into CHO (ATCC, Manassas, VA) cells using a patented Artificial Chromosome Expression (ACE) system [21]. Cell lines were transferred to Goodwin Biotechnology (Plantation, FL) for the development of master cell banks and the production and purification of the 2556 mAb. All activities at Goodwin Biotechnology were conducted under cGMP. The clinical grade 2556 mAb produced by this methodology was used in all described below in vitro and in vivo experiments.

### Immunoreactivity and radiolabeling of 2556

Immunoreactivity of clinical grade 2556 towards gp41 antigen was compared to that of heterohybridoma produced mAb and 246 mAb utilized in our previous RIT of HIV report [14] by standard gp41-binding ELISA. Gp41_MN_ protein was purchased from Immunodiagnostics (Woburn, MA) and gp41_IIIB_ - from Virogen (Watertown, MA). For radiolabeling 2556 mAb was conjugated to the chelating agent *C*-functionalized *trans*-cyclohexyldiethylene-triamine pentaacetic acid derivative (CHXA″) (Macrocyclics, San Antonio, TX) as described in [14] using 2–20 initial molar excess of the chelating agent over mAb. The purification of conjugated 2556 from the unreacted chelating agent was accomplished using Amicon microfiltration system. ^213^Bi was obtained from the NorthStar NM ARS II automated ^225^Ac/^213^Bi radionuclide generator system (Janesville, WI). ^225^Ac parent for ARS II was procured from Oak Ridge National Laboratory (Oak Ridge, TN). Radiolabeling of 2556 with ^213^Bi was performed as in [14]. Serum stability of ^213^Bi-2556 in human serum was assessed for 3 hr at 37°C by instant thin layer chromatography (ITLC) in 0.15 M ammonium acetate buffer. In this system labeled proteins stay at the bottom while ^213^Bi in form of small complexes such as ^213^Bi-CHXA″ (if it would separate from the antibody) moves with the solvent front.

### HIV-1 infected cell lines

We utilized both chronically and acutely HIV-1 infected human cell lines in the study. The ACH-2 cell line obtained through the NIH AIDS Research and Reference Reagent Program (Division of AIDS, NIAID, NIH: ACH-2, catalogue #349 from Dr. Thomas Folks) is a latent T-cell clone infected with HIV-IIIB that produces steady low levels of viruses that is markedly increased by stimulation with phorbol myristate (PMA). The ACH-2 parental cell line A3.01 (NIH AIDS Research and Reference Reagent Program catalogue # 166), which is HIV-1-negative, was used as a control. For acute infection hPBMCs obtained from New York Blood Center (NY, NY) were utilized. hPBMCs were stimulated with phytohemaglutinin (PHA) and IL-2 for 48 h and then infected with HIV-1 JR-CSF (NIH AIDS Research and Reference Reagent Program, NIAID, NIH: HIV-1_JR-CSF_, catalogue #394 from Dr. Irvin Chen). While the number of ACH-2 cells infected with HIV-1 was almost 100%, only a fraction (∼10%) of the hPBMCs were infected with HIV-1 as determined by limiting dilution co-culture technique [14], [22]. Further in the text we refer to the cells exposed to HIV-1 as “infected” cells and those which were not exposed to the virus as “non-infected” cells.

### Binding of 2556 to HIV-1 chronically-infected ACH-2 cells

The abilities of mAbs 2556 and 246D, and control mAb 1418 to bind specifically to HIV-1 expressed by chronically-infected ACH-2 cells, stimulated for 48 h with 0.1 µM phorbol myristate acetate (PMA) (Sigma) in RPMI-1640 containing 10% fetal calf serum (FCS) were determined by flow cytometry. Cells were stained with either the mAbs 2556, 246D, or 1418 for 30 min at 4°C, washed and stained with a secondary goat F(ab′)_2_ anti-human IgG conjugated to phycoerythrin (Invitrogen), for 30 min at 4°C. Cells were analyzed using a Coulter FC500 flow cytometery and FlowJo software.

### Scatchard analysis of ^213^Bi-labeled 2556 binding to the HIV1-infected hPBMCs

Scatchard analysis of ^213^Bi–labeled mAbs 2556 and 246D binding to infected hPBMCs was performed with 2×10^6^ hPBMCs per sample. The percentage of gp41 expressing cells was assumed to be 10%. MAbs were added to the samples in 0.12–0.48 nM concentrations. After incubation for 1 hr at 37°C, the tubes were counted in a gamma counter, the cells were collected by centrifugation and the pellets were counted again. Scatchard analysis was used to compute the mAb binding constant (K_a_) and binding sites per cell as previously described [23].

### *In vitro* killing of ACH-2 cells and hPBMCs with radiolabeled mAbs

For in vitro killing experiments ACH-2 cells were stimulated with 0.1 µM PMA for 48 h, and then stimulated or non-stimulated ACH-2 cells in triplicate wells were treated with 5–20 µCi ^213^Bi-labeled 2556 or control 1418 mAb or with matching amounts (2.5–10 µg) of “cold” 2556 mAb. Approximately 2×10^5^ cells were used for each condition. The cells were incubated with radiolabeled or “cold” mAbs at 37°C for 3 h, transferred into fresh cell culture medium and then incubated in 5% CO_2_ at 37°C for 72 h. The number of viable cells was then assessed by trypan blue dye exclusion assay. For experiments with hPBMCs the cells were used 48 h after infection with HIV-1 JR-CSF and the killing assay was conducted as described above.

### In vivo evaluation of ^213^Bi-2556 in HIV-1 mouse models

All animal experiments were performed at the University of California, Los Angeles, Medical Center and were fully approved by IACUC and by the UCLA Animal Research Committee (ARC) in accordance with all federal, state, and local guidelines under AAALAC #000408 and ARC protocol #1996-058-51. We employed two HIV-1 mouse models – splenic model [14] and peritoneal model [24]–[26] to conduct the evaluation of ^213^Bi-2556 ability to kill HIV-1 infected PBMCs in vivo as well as its potential toxicity. MAb 1418 was used as a non-specific control.

#### Splenic model

Human PBMCs utilized in the mouse studies were obtained from the UCLA Center for AIDS Research (CFAR) Virology Core Laboratory and were stimulated with 5 µg/mL PHA and 20 units/mL interleukin(IL)-2 in RPMI-1640 containing 10% FCS for 3 days. The cells were then infected with the CCR5-tropic HIV-1 strain JR-CSF, a gift of Dr. Irvin Chen. Prkdc−/− C.B. 17 severe combined immunodeficient (SCID) mice were bred by the Division of Laboratory Medicine (DLAM) at UCLA. Four days post-infection, infected hPBMCs were injected intrasplenically (2.5×10^7^ cells per mouse) into SCID mice. Mice were treated intraperitoneally (i.p.) 1.5 hr later with one of the following: cold 2556; 50, 100 or 200 µCi of either ^213^Bi-2556 or ^213^Bi-1418; saline (n = 8 per group). Mice were sacrificed 3 d later and spleens were harvested and processed. Mice in 200 µCi of either ^213^Bi-2556 or ^213^Bi-1418 mAbs were assessed only for acute hematologic toxicity by peripheral blood platelet counts. In the rest of the groups viral load was measured by quantitative real-time polymerase chain reaction (RT-PCR) of HIV-1 DNA as previously described [27]. In addition, the number of HIV-1-infected cells present in the spleen was measured using limiting dilution quantitative co-culture as described in [22]. Mouse peripheral blood platelet counts were determined by the UCLA DLAM clinical laboratory.

#### Peritoneal model

Ten-twenty million hPBMCs (UCLA CFAR Virology Core) were implanted into SCID mice by i.p. injection as previously described [25], [26]. Fourteen days post-implantation, hPBMC SCID mice were infected with HIV-1 JR-CSF via i.p. injection. Two days post-infection, the mice were treated i.p. with one of the following: 25, 50 or 100 µCi of either ^213^Bi-2556 or ^213^Bi-1418; or saline (n = 3–6 per group). Mice were sacrificed 3 d later and peritoneal lavage was performed to recover hPBMCs. Viral load was determined by quantitative real-time PCR of HIV-1 DNA [27] in SCID-derived human cells; and mouse peripheral blood was analyzed for platelet counts. Two mice per group were kept for additional 10 d and their gross pathology was evaluated after sacrifice. Mouse peripheral blood platelet counts and gross pathology were performed by the UCLA DLAM clinical laboratory.

## Results

### Initial selection and characterization of 2556 mAb

**Figure 1:**
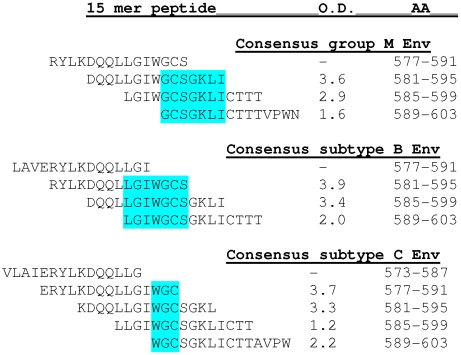
Binding of mAb 2556 to 15-mer overlapping gp41 peptides. The common sequence which 2556 reacts with the various overlapping peptides and corresponding OD values are labeled blue. MAb 2556 can bind to a linear epitope which is located in the region of the N-terminal cysteine (AA 591) of the immunodominant disulfide loop of gp41. The 2556 epitope contains two residues GC (bold and underlined) which are recognized by the mAb in all sequences (core epitope) and few residues in the vicinity which contribute to this epitope.

**Figure 2:**
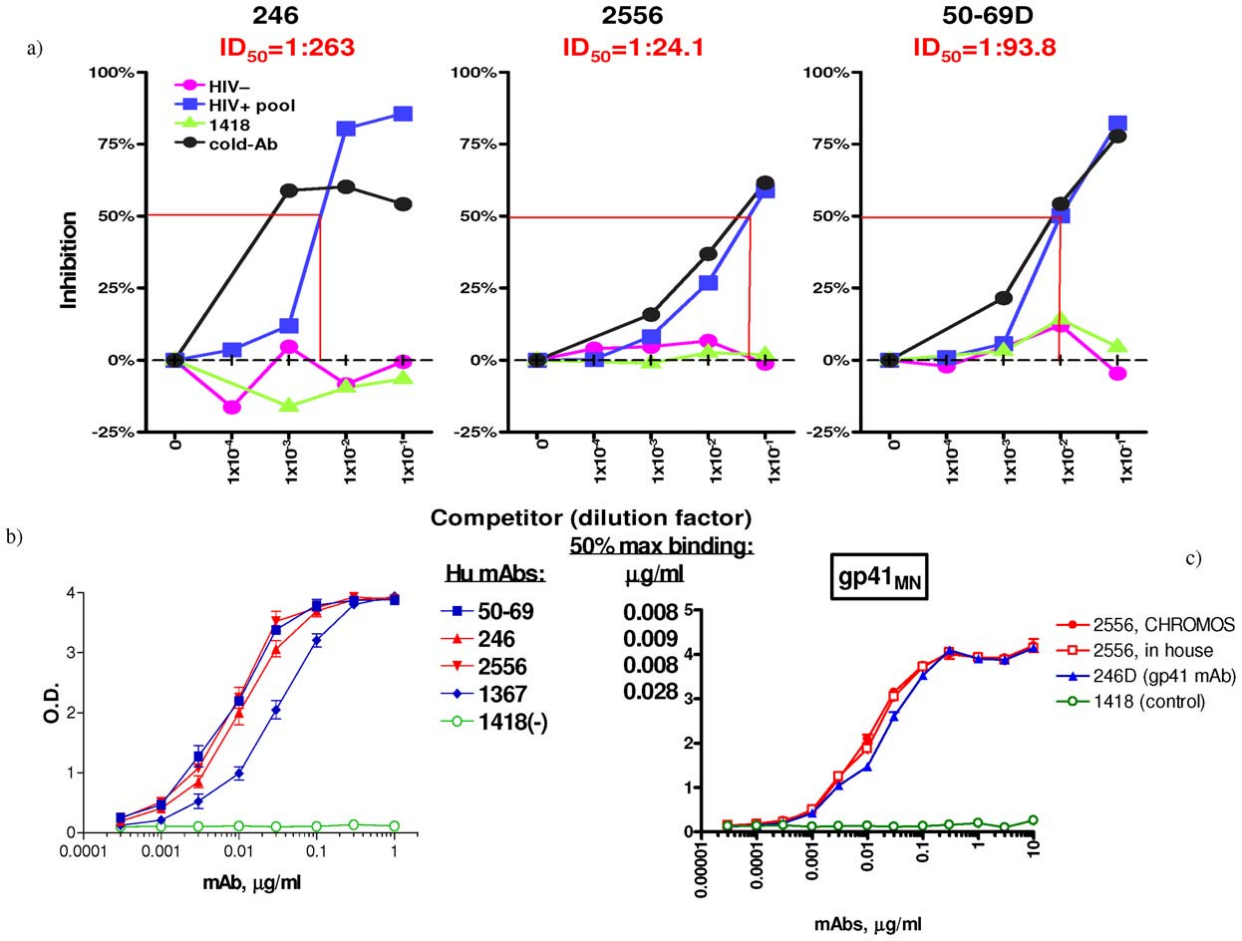
Characterization of 2556 mAb specificity and selectivity. a) inhibition of binding of biotinylated anti-gp41 mAbs to JR-CSF transfected 293T cells by serum from HIV-1 infected individuals; b) binding of human anti-gp41 mAbs to recombinant gp41_MN_ protein; c) ELISA reactivity to recombinant gp41_MN_ of 2556 CHROMOS (made by ACE system) and 2556 (in house) derived from original NYU hybridoma. 246D is an anti-gp41 mAb and 1418 is a negative control.

The mAb 2556 epitope was mapped by defining the common sequence recognized by mAb reactivity to the 15-mer gp41 peptides representing three sequences of group M, subtype B and C, and which ovelapped by 11 amino acids. This study showed that mAb 2556 can bind to a linear epitope which is located in the region of the N-terminal cysteine (AA 591) of the immunodominant disulfide loop of gp41 (Fig. 1). The mAb 2556 epitope contains two residues, Glycine (G) and Cysteine (C), which are recognized by mAb 2556 in all reactive sequences (core epitope) and few residues in the close vicinity which contribute to the binding. MAbs 246 and 50–69 were more effectively competed by patients' sera than mAb 2556 in that the former were inhibited by 50% at higher serum dilutions. For example, the serum dilution for 50% inhibition (ID_50_) of biotinylated mAbs 246, 2556 and 50–69 for binding to JR-CSF-infected cells was 1∶263, 1∶24.1 and 1∶93.8, respectively (Fig. 2a). Similar results were obtained when the mAbs were tested against cells expressing a clade AG Env (DJ263) and clade C Env (MW965) (data not shown). The comparative study with other anti-gp41 mAbs showed that mAb 2556 had the same relative affinity as mAbs 50–69 and 246, because the same concentration of each was needed for half-maximal binding to gp41. The relative affinity, 0.008 µg/ml, corresponds to affinities in the nanomolar range (0.1 nM) (Fig. 2b).

The binding activity of mAb 2556 to infected cells and intact virions was comparable to the two other anti-gp41 mAbs while the negative control, mAb 1418, was not reactive. This experiment confirmed that mAb 2556 reacts with all tested viruses from different HIV-1 subtypes expressed on the surface of transfected 293T cells. MAb 2556 was also cross-reactive with intact infectious HIV-1 viruses. MAb 2556 and two other anti-gp41 mAbs bound to all 42 intact virions with their binding activity above background as determined using mAb 1418 as negative control (data not shown).

### Immunoreactivity and radiolabeling

**Figure 3:**
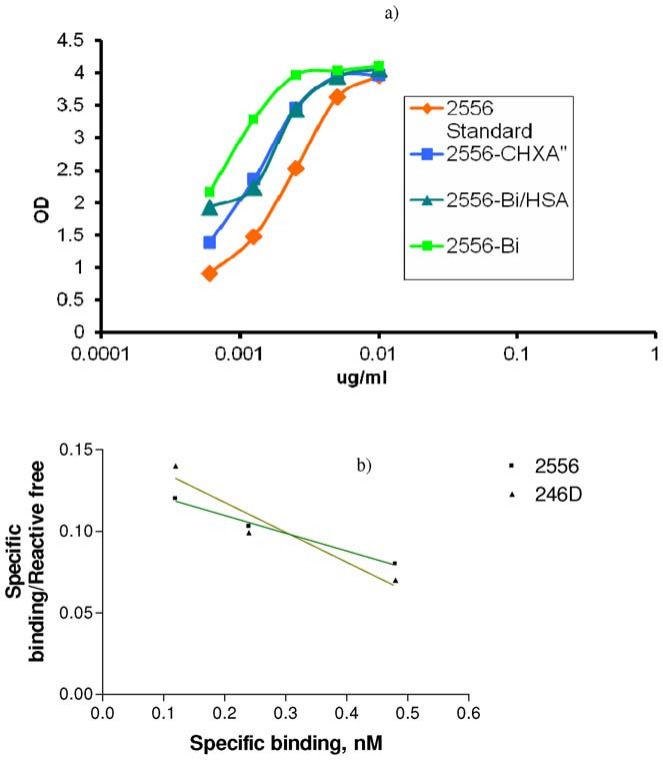
Immunoreactivity of ^213^Bi-2556 mAb for gp41 and determination of its K_a_ by Scatchard analysis. a) gp41 ELISA of 2556 mAb radiolabeled with high specific activity ^213^Bi (5 mCi/mg). Binding of ^213^Bi-2556 to gp41 was compared to that of unlabeled 2556 mAb with (2556-CHXA″) or without (2556 standard) attached CHXA″ ligand. ^213^Bi-2556 was stored with human serum albumin (HAS) radioprotector (2556-Bi/HAS) or without it (2556-Bi); b) Scatchard plot of ^213^Bi-labeled 2556 and 246D mAbs binding to HIV-infected hPBMCs.

The clinical lot mAb 2556 produced from CHO cells (CHROMOS/ACE system) bound gp41 antigen by ELISA in the same way as mAb 2556 derived from the original hetero-hybridoma (Fig. 2c). The attachment of CHXA″ chelating agent with the initial chelating agent to protein molar ratios up to 20 did not decrease the immunoreactivity of mAb 2556. Fig. 3a displays the ELISA of unlabeled CHXA″-conjugated mAb 2556 (initial molar excess of 2) and the same batch radiolabeled with ^213^Bi and incubated at 37°C with and without human serum albumin (HSA) as a radioprotector. It is clear that conjugation and radiolabeling did not result in decrease of mAb 2556 binding to gp41 antigen and that even at the high specific activity of 5 mCi/mg, the radiolabeled mAb 2556 preserved its immunoreactivity for 3 hrs without the need for a radioprotector. The radiolabeling purity of various batches of ^213^Bi-2556 was 90–95% which did not require post-labeling purification. Serum stability experiments showed that 93% of ^213^Bi-2556 was intact in human serum at 1 hr incubation, 94% - at 2 hr and 93% - at 3 hr which permitted us to conclude that ^213^Bi-2556 was stable in serum. In addition it should be noted that because of the ^213^Bi short physical half-life of 46 min at 3 h only 6% of ^213^Bi activity remains.

### Scatchard analysis

Scatchard analysis of ^213^Bi-2556 and ^213^Bi-246D binding to HIV-1_JR-CSF_ infected hPBMCs (Fig. 3b) allowed us to calculate K_a_ and estimate the number of binding sites for both mAbs as follows: K_a_ for ^213^Bi-246D 1.8×10^8^ M^−1^ and 2.5×10^6^ binding sites per cell; K_a_ for ^213^Bi -2556 1.1×10^8^ M^−1^ and 3.6×10^6^ binding sites per cells. These data attest to the high affinity of 2556 for gp41 antigen and high number of binding sites on HIV-1 infected cells.

### In vitro killing of ACH-2 and infected hPBMCs with ^213^Bi-2556

**Figure 4:**
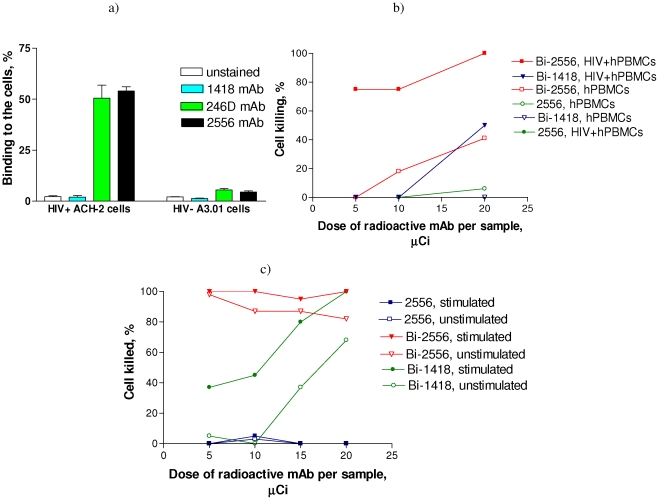
Targeting and killing of HIV-infected cells in vitro with ^213^Bi-2556 mAb. a) binding of “cold” 2556 to chronically-infected ACH-2 cells by flow cytometry. HIV-1-negative A3.01 cells were used as controls; b) killing of HIV-infected hPBMCs with ^213^Bi-2556 mAb. 246D is an anti-gp41 mAb and 1418 is a negative control; c) killing of chronically-infected ACH-2 cells with ^213^Bi-2556 mAb. Stimulated - PMA stimulated ACH-2 cells, unstimulated – unstimulated ACH-2 cells. The unlabeled antibodies were present in the same amounts as the radiolabeled antibodies.

Flow cytometry experiments ascertained specific binding (up to 60%) of mAb 2556 to chronically infected, activated ACH-2 cells and almost none to control, activated A3.01 cells (Fig. 4a). Both PMA-stimulated and non-stimulated cells were completely killed in the total range of ^213^Bi-2556 activities used in the experiment while killing with ^213^Bi-1418 control mAb was significantly less and approached that of ^213^Bi-2556 only for the highest dose per sample (Fig. 4b). Such killing with non-specific mAb is due to high concentration of radioactivity in a well when cells are killed by random “cross-fire” radiation and has no physiological relevance where only bound mAbs are capable of delivering the radiation to the cells. No killing of cells with “cold” mAb 2556 was observed (Fig. 4b). A similar trend was observed for infected and non-infected hPBMCs (Fig. 4c) with killing of infected hPBMCs with ^213^Bi-2556 significantly higher than that of controls.

### Efficacy and safety of ^213^Bi-2556 in splenic and peritoneal HIV-1 mouse models

**Figure 5:**
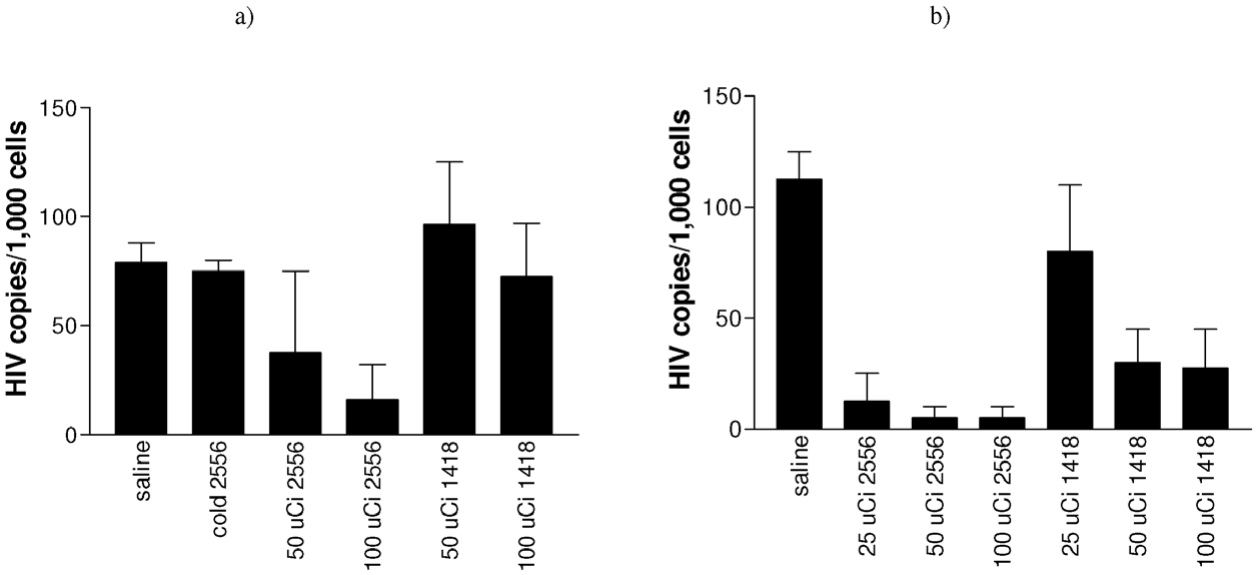
RT-PCR data for two HIV-1 mouse models used in RIT experiments with ^213^Bi-2556 mAb. a) splenic model (mice given HIV-1 infected hPBMCs intrasplenically); b) peritoneal model (mice given non-infected hPBMCs followed by i.p. challenge with HIV). “Cold” 2556 – unlabeled 2556; 1418 – isotype-matching irrelevant control.

To examine the ability of ^213^Bi-2556 to kill HIV infected cells *in vivo*, we initiated the testing of ^213^Bi-2556 mAb in splenic mouse model of HIV-1 infection (Fig. 5a) in a manner similar to that previously described [14]. The PCR-based analysis for HIV viral load in the recovered human cells derived from treated mice hPBMCs demonstrated that ^213^Bi-2556 was more effective in killing the HIV-1 infected hPBMCs than the non-HIV specific control mAb labeled with the same amounts of radioactivity or unlabeled 2556 (P<0.05) (Fig. 5a). The elimination of infected hPBMCs was dose dependent with the most pronounced killing of hPBMCs observed in 100 µCi ^213^Bi-2556 group (P = 0.01). The PCR results were confirmed by the limiting dilution co-culture method (Fig. S1). Further evaluation of ^213^Bi-2556 efficacy and safety was performed utilizing the hPBMC SCID peritoneal model of HIV-1 infection, which allows greater HIV infection and spread in vivo than the splenic model and is more physiologically relevant for assessment of killing of virally infected cells than a splenic model. Lower doses of radiolabeled mAbs were used in this study to gauge further the dose response in killing of infected hPBMCs. There was an order of magnitude reduction in viral load in groups treated with ^213^Bi-2556 as compared to all controls (P<0.05) (Fig. 5b) with 50 and 100 µCi doses being equally effective in eliminating infected hPBMCs with the number of HIV copies per 1,000 cells in these two groups approaching the level of assay sensitivity (3 copies of HIV per 1000 cells).

**Figure 6:**
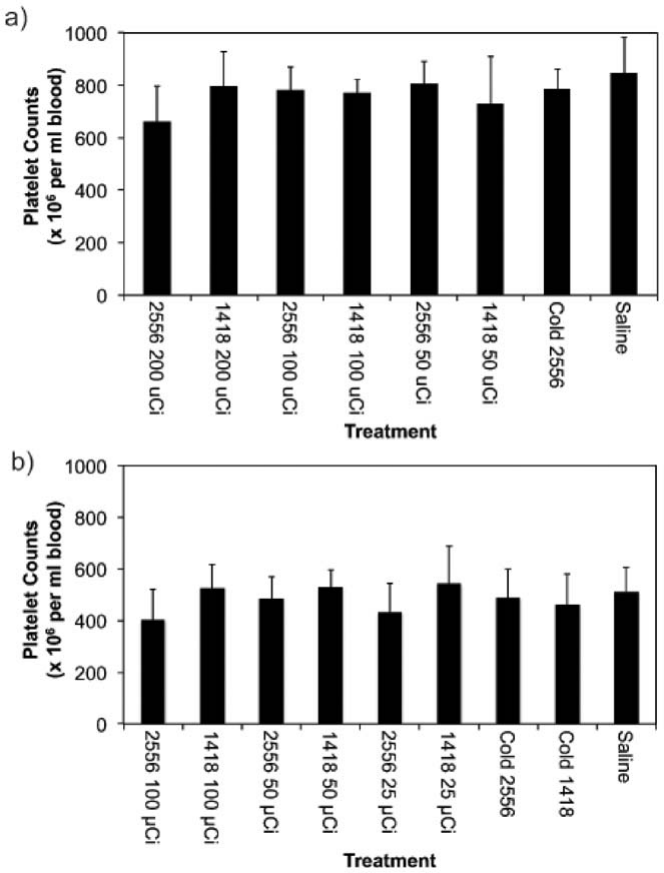
Peripheral blood platelet counts from SCID mice used in (a) splenic and (b) peritoneal HIV models and treated with ^213^Bi-2556 and control mAbs.

For safety evaluation platelet counts in mice treated with radiolabeled mAbs were measured. In the splenic model there were no significant differences in platelet counts between treated and control mice even for the highest 200 µCi dose pointing to the absence of acute hematologic toxicity of RIT (P>0.05) (Fig. 6a). Likewise, in the peritoneal model both platelet counts (Fig. 6b) and the results of gross pathology evaluation (not shown) showed the absence of toxic effects of RIT towards bone marrow when compared to non-radioactive controls (P>0.05) and all major organs, respectively. Thus, in both studies treatment with ^213^Bi-2556 resulted in the significant reduction of HIV infection in vivo in a highly effective and biologically safe manner.

## Discussion

In cancer treatment, the success of FDA-approved drugs such as Zevalin® and Bexxar® (anti-CD20 mAbs labeled with 90-Yttrium (^90^Y) and 131-Iodine (^131^I), respectively), in the treatment of primary, relapsed or refractory B-cell NHL administered as a single dose is evidence of the enormous potential of RIT for targeted elimination of malignant cells. This clinical experience using RIT creates a favorable environment for the introduction and use of additional RIT approaches, such as developing RIT for treating HIV-1 infected patients. The effectiveness of RIT for HIV-1 infection is enhanced because the majority of long-lived infected cellular targets are lymphocytes, which are among the most radiosensitive cells in the body.

Based on our encouraging proof-of-principle results targeting HIV-1 infected cells in vitro and in vivo with radiolabeled mAb 246D to gp41 viral antigen [14], we embarked on identifying the best candidate mAb for a preclinical development. For this purpose we performed initial selection and characterization of 2556, the evaluation of clinical grade 2556 in vitro, and the efficacy and safety studies of clinical grade 2556 radiolabeled with α-emitter ^213^Bi in HIV-1 mouse models. The best mAb to use for RIT is a mAb that binds to the immunodominant domain (cluster I) of gp41 which is displayed on infected cells. Among the several anti-gp41 human mAbs tested, one mAb was found to display the characteristics that would make it the favored candidate for use as an RIT reagent: mAb 2556 displayed high binding affinity, reacted with multimeric forms of gp41 present on both the surface of virus particles and cells expressing HIV-1 Env, and recognized a highly conserved epitope of gp41 shared by all HIV-1 subtypes which is accessible for antibody binding both on virus-transfected cells and on intact infectious viruses. Also, in comparison to other anti-gp41 mAbs, mAb 2556 was best able to compete with HIV-1+ serum antibodies which is extremely important for efficacy in the treatment of HIV-1-infected subjects. This is a very important consideration because the presence of these antibodies could conceivably defeat RIT by blocking the binding site of the therapeutic antibody. In this regard, the study of gp41 mAbs binding to 26 intact primary virions of clade A to H showed that gp41 cluster I mAbs bound strongly across all the HIV-1 clades examined while mAbs to cluster II bound poorly or sporadically to the isolates [28]. In particular, two gp41 cluster I mAbs, 246-D and 240-D, directed at core epitopes LLGI and IWG, respectively, located at the apex of this hydrophilic immunodominant region, bound better than any other gp41 mAbs tested [28]. Based on the competition assay mAb 2556 recognizes the same epitope as mAbs 246-D and 240-D and binds similarly well to intact virions and for these reasons was selected for radiolabeling and RIT experiments. There are several more gp41 epitopes recognized by human anti-gp41 mAbs as recently reported [29] however they are not immunogenic possibly due to non-covalent association of gp120 with gp41 which shields the latter. The 2556 epitope is well exposed, as epitopes of 246-D and 240-D, and radiolabelled 2556 have good access to the infected cells expressing virus envelope proteins on the surface. The access to virus envelope is critical for efficient RIT and some competition with serum anti-gp41 antibodies can be overcome eventually by testing different doses of the compound which will be determined in the clinical trial.

We chose ^213^Bi as a radionuclide for labeling of 2556 mAb not only because it proved to be effective in elimination of HIV-1 infected cells in our previous studies but also because it has shown to be effective and safe in treatment of various malignancies in cancer patients [30]–[32]. MAb 2556 was robust towards attachment of ligand CHXA″ required for further radiolabeling with ^213^Bi. Radiolabeled 2556 had high affinity for its gp41 antigen according to the Scatchard analysis. High affinity is a pre-requisite for a successful RIT as its efficacy is directly proportional to a mAb affinity for its respective antigen [33]. Furthermore, the finding that there are more than 10^6^ binding sites for mAb 2556 on the surface of infected hPBMCs will also contribute to the RIT efficacy as it is known from cancer RIT that successful killing of cells with radiolabeled mAbs requires approximately 10^5^ binding sites per cell. These two factors contributed to the elimination of both PMA-stimulated and non-stimulated ACH-2 cells with ^213^Bi-2556 as even very moderate expression of gp41 on non-stimulated ACH-2 cells is sufficient for a high affinity mAb to deliver cytocidal doses of radiation to the cells. In addition, the latently-infected cells located in close proximity to cells targeted with the radiolabeled mAb will be killed by “cross-fire” radiation - the killing of cells by the radiation emanating from the antibody bound to the adjacent or distant cells.

Initial testing of ^213^Bi-2556 in the splenic HIV-1 SCID mouse model demonstrated dose-dependent specific killing of infected hPBMCs and lack of acute hematologic toxicity. Further evaluation was performed in more physiologically relevant peritoneal model of HIV-1 developed by D. Mosier [24]–[26]. This model involves implantation of human PBMCs into SCID mice by i.p. injection that results in T-cell activation, provides a ready target for HIV-1 infection, and mimics the extensive lymphocyte activation seen in chronic HIV-1 infection. HIV-1 infection of hPBMC SCID mice leads to loss of CD4+ T cells, the primary consequence of HIV-1 infection in humans. This mouse model has been used extensively in evaluating efficacy of anti-viral agents [34], [35]. Again, the efficacy of ^213^Bi-2556, now administered in the lower dose range of 25–100 µCi, in comparison with all controls was obvious and statistically significant (P<0.05). It should be noted that the dose of 100 µCi in a 20 g mouse is equal to the dose of 28.5 mCi in a 70 kg human when the difference between the body surface to body weight ratios (0.0066 m^2^/0.02 kg for a mouse and 1.6 m^2^/60 kg for a human [36]) is taken into consideration. Such doses have been shown to be effective and safe in treatment of myeloid leukemia in patients with ^213^Bi-labeled mAbs [30].

RIT of HIV-1 has several advantages over the immunotoxin approach: 1) The Ab used for radiation delivery does not need to be internalized to kill the cell; 2) Not every infected cell in the body needs to be targeted by the antibody because of the “cross-fire” effect. 3) In contrast to immunotoxins, the radioisotope linked to the antibody is unlikely to elicit significant immune responses that would limit subsequent use; 4) RIT is potentially less toxic since the chemistry of linking different radioisotopes to the antibodies has been well developed and the exceptional stability of radiolabeled mAbs in vitro and in vivo has been confirmed [30]–[32]. 5) The availability of many isotopes differing in half-life and radiation type offers great versatility for designing RIT.

The experiments described in the paper demonstrate the potential of RIT to eradicate HIV-infected cells when they are actively producing the virus and expressing gp-41 antigen on their surface. These results are encouraging as they demonstrate that RIT might be used in patients with active HIV disease, and performing a clinical trial in such patients represents one possibility of investigating RIT efficacy in humans. Unfortunately, it is difficult to rely on existing macaque models such as SIV to test RIT efficacy before transitioning into humans as according to Berger and Pastan [6] it is presently unclear if those models faithfully replicate the mechanisms of HIV persistence in humans. Besides testing RIT strategy in patients with active HIV disease, another cohort of patients would be those on HAART with suppressed plasma viremia. As suggested in [6] which discusses immunotoxins application for HIV eradication, such trial could test 2-tier (HAART and RIT) or 3-tier (HAART, treatment to trigger the activation of latently infected cells and RIT) strategies. The read-out for a RIT trial in any patient population should be analysis of the peripheral blood for proviral DNA and infectious virus in CD4+ T cells as well as gut biopsy to analyze the gut-associated lymphoid tissue (GALT) for HIV presence.

In conclusion, we describe the preclinical development of a novel RIT reagent which will be a part of HIV eradication strategy ready for translation into the clinic as the next step. As viral antigens are very different from “self” human antigens - this approach promises high specificity of the treatment, which should result in increased efficacy and low toxicity. These features are particularly attractive for HIV-1 infected patients whose immune status and bone marrow reserves are very low.

## Supporting Information

Figure S1 **Limiting dilution co-culture results on evaluating the efficacy of ^213^Bi-2556 mAb in HIV-1 mouse splenic model.** 1418 mAb was used as an irrelevant isotype-matching control.
(TIF)
