# Correction: Pre-Clinical Evaluation of a ^213^Bi-Labeled 2556 Antibody to HIV-1 gp41 Glycoprotein in HIV-1 Mouse Models as a Reagent for HIV Eradication

**DOI:** 10.1371/annotation/c4cb1c2c-a436-4f7a-ada9-1feb42aebca1

**Published:** 2012-08-07

**Authors:** Ekaterina Dadachova, Scott G. Kitchen, Gregory Bristol, Gayle Cocita Baldwin, Ekaterina Revskaya, Cyril Empig, George B. Thornton, Miroslaw K. Gorny, Susan Zolla-Pazner, Arturo Casadevall

There was information missing from the funding section. The complete, correct funding statement is: This study was funded by Pain Therapeutics who, at the time of the study, had the license for RIT of HIV. The funders paid for research personnel effort, supplies and laboratory animals through industrial research agreements with Albert Einstein College of Medicine and with University of California Los Angeles. Currently Pain Therapeutics do not have the licensing rights for this technology. This work was also supported in part by the Bill and Melinda Gates Foundation (Grand Challenge Exploration grant OPP1035945 to ED) and Center for AIDS Research at the Albert Einstein College of Medicine and Montefiore Medical Center funded by the National Institutes of Health (NIH AI-51519). Pain Therapeutics participated in the study design, analysis, decision to publish, and preparation of the manuscript. Other funders did not participate in the study design, analysis, decision to publish, and preparation of the manuscript. 

