# Supplementary figures and images for: Pre-Clinical Evaluation of a 213Bi-Labeled 2556 Antibody to HIV-1 gp41 Glycoprotein in HIV-1 Mouse Models as a Reagent for HIV Eradication
Source: PLoS One. 2012 Mar 9;7(3):e31866. doi: 10.1371/journal.pone.0031866 (PMC3302885; doi:10.1371/journal.pone.0031866)

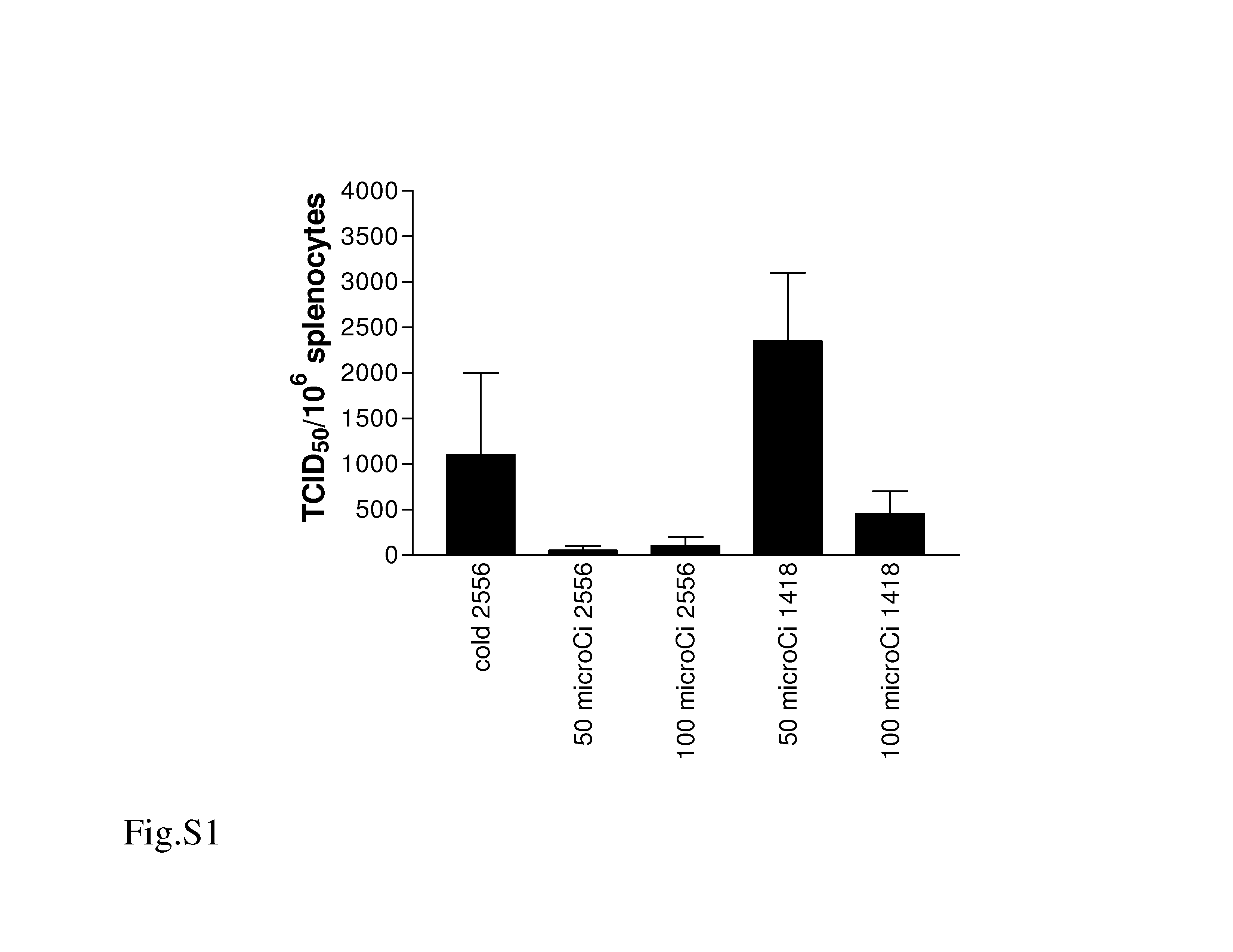

Supplement: Figure S1 — Limiting dilution co-culture results on evaluating the efficacy of 213Bi-2556 mAb in HIV-1 mouse splenic model. 1418 mAb was used as an irrelevant isotype-matching control. (TIF) [file pone.0031866.s001.tif]
